# Chronic Recurrent Multifocal Osteomyelitis Associated with Crohn Disease: A Potential Role of Exclusion Diet? Comment on Starz et al. The Modification of the Gut Microbiota via Selected Specific Diets in Patients with Crohn’s Disease. *Nutrients* 2021, *13*, 2125

**DOI:** 10.3390/nu13114005

**Published:** 2021-11-10

**Authors:** Erika Cantarelli, Francesco Baccelli, Gabriele Simonini, Patrizia Alvisi

**Affiliations:** 1 Specialty School of Pediatrics-Alma Mater Studiorum, University of Bologna, 40138 Bologna, Italy; erika.cantarelli@studio.unibo.it (E.C.); francesco.baccelli2@studio.unibo.it (F.B.); 2 Department of Pediatric Rheumatology, Meyer Children University Hospital, 50139 Florence, Italy; gabriele.simonini@unifi.it; 3 Pediatric Gastroenterology Unit, Maggiore Hospital, Azienda USL, 40133 Bologna, Italy

**Keywords:** resistant Crohn disease, chronic recurrent multifocal osteomyelitis, Crohn disease exclusion diet

## Abstract

The efficacy of diet and its influence on gut microbiome composition has been largely demonstrated in inflammatory bowel disease (IBD). Little is known about its potential in the management of extraintestinal manifestations. We report a successful application of Crohn disease exclusion diet (CDED) in association with infliximab and methotrexate, as salvage therapy in a child affected by chronic recurrent multifocal osteomyelitis (CRMO) and Crohn disease (CD) resistant to optimized therapy. Both intestinal and bone symptoms remitted after the application of CDED. Diet may have acted on common microbic inciting agents that trigger both intestinal and bone inflammation, supporting the role of microbiota in the pathogenesis of IBD-associated extraintestinal manifestations. Our experience suggests the potential benefit of CDED in association with combined therapy in resistant patients affected by CD and extraintestinal manifestations.

We read with interest the publication by Starz and colleagues [1] regarding the association of nutritional therapies and gut microbiome modifications in inflammatory bowel diseases (IBD). The efficacy of diet has been demonstrated in IBD [2]. Little is known about its potential in the management of extraintestinal manifestations. We report a successful application of Crohn disease exclusion diet (CDED) in association with infliximab and methotrexate, as rescue therapy in a child affected by chronic recurrent multifocal osteomyelitis (CRMO) and Crohn disease (CD) resistant to optimized therapy. A 10-year-old boy complaining of migrating bone pain was diagnosed with CRMO by MRI and bone biopsy. Adalimumab was started with uncompleted clinical remission. Two months later, he developed bloody diarrhea and weight loss with elevated CRP and fecal calprotectin. Colonic CD with mild activity was diagnosed by endoscopy. Adalimumab was implemented according to pediatric guidelines [3]. Because of persistence of both intestinal and rheumatological manifestations, methotrexate was added. The patient did not clinically respond, and colonoscopy revealed the extent of disease to be pancolitis with moderate activity. Adalimumab was switched to infliximab. Clinical response was not achieved after induction phase, and infliximab therapy was optimized without success. We therefore introduced CDED plus partial enteral nutrition (PEN), as suggested by Levine et al. [4]. At the end of the first diet phase, clinical remission was eventually obtained for both rheumatological and intestinal symptoms with significant weight gain. Calprotectin and CRP values normalized. Mucosal healing was documented. At present, the patient exhibits stable disease control and undergoes therapy with infliximab and methotrexate associated with the third diet phase. The clinical course is highlighted in Figure 1.

This is the first reported case of CRMO associated with CD in which CDED has been attempted. Both intestinal and bone symptoms were resistant to biologics associated with immunomodulator, even after optimization. Remission was achieved after application of CDED. In recent years, the efficacy of exclusion diet plus PEN in inducing sustained remission in CD pediatric patients has been demonstrated, even in subjects resistant to biologics [4,5]. Similarly to exclusive enteral nutrition, CDED demonstrated an intestinal anti-inflammatory effect with a mechanism based on the exclusion of foods that alter microbiota [4], which have a principal role in CD pathogenesis [2]. In our case, CDED may have acted on common microbic inciting agents that trigger both intestinal and bone inflammation. Protection against osteomyelitis by diet-induced changes of intestinal microbiome has been observed in CRMO murine models [6]. These findings suggest the role of microbiota in the pathogenesis of IBD-associated extraintestinal manifestations.

In conclusion, our experience suggests the potential benefit of CDED in association with combined therapy in resistant patients affected by CD and extra-intestinal diseases such as CRMO. Further studies are needed to better understand the role of nutritional strategies in these conditions. 

## Figures and Tables

**Figure 1:**
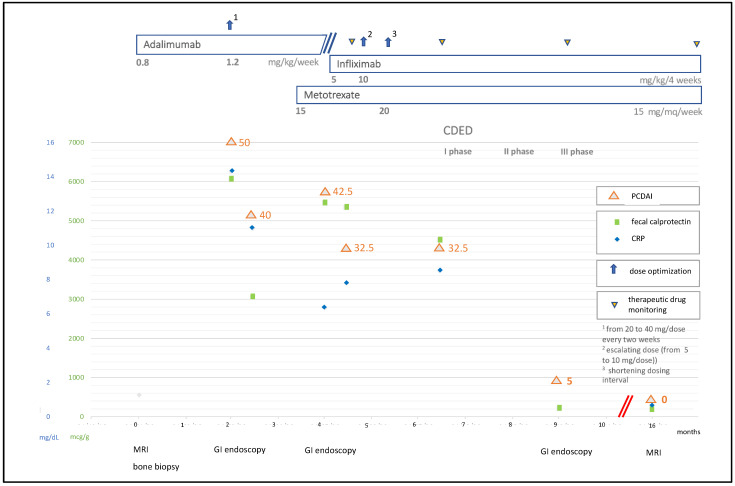
Clinical, laboratory, and endoscopic evolution together with treatments adopted at different time points. CDED: Crohn disease exclusion diet; PDCAI: pediatric Crohn’s disease activity index; CRP: C-reactive protein; MRI: Magnetic Resonance Imaging; GI: gastrointestinal.
